# Prevalence of induced abortion among Chinese women aged 18–49 years: Findings from three cross-sectional studies

**DOI:** 10.3389/fpubh.2022.926246

**Published:** 2022-10-03

**Authors:** Liangyu Kang, Jue Liu, Qiuyue Ma, Wenzhan Jing, Yu Wu, Shikun Zhang, Min Liu

**Affiliations:** ^1^Department of Epidemiology and Biostatistics, School of Public Health, Peking University, Beijing, China; ^2^Chinese Association for Maternal and Child Health Studies, Beijing, China

**Keywords:** induced abortion, childbearing age, women's health, reproductive health, China

## Abstract

There are few latest researches about induced abortion in China. We aimed to evaluate the prevalence of induced abortion and the related factors, thereby helping make targeted policies and measures to promote women's health. Three comparable cross-sectional surveys among Chinese women aged 18–49 years were performed in 2016, 2017, and 2021. A total of 14,573 eligible respondents were included in the study. 16.70% (95%CI 16.10%-17.31%) of respondents self-reported having experienced induced abortion, while 6.88% (95%CI 6.46%-7.29%) self-reported repeat induced abortion. Age range of 25–49 years (aOR 2.27–6.31, all *P*<0.05), living in western (aOR 1.72, 95%CI 1.50–1.98) and central (aOR 1.36, 95%CI 1.21–1.52) regions, having children (aOR 2.85, 95%CI 2.35–3.46) were associated with higher prevalence of induced abortion. Moreover, age range of 25–49 years, living in western and central regions, having children were also related to higher prevalence of repeat induced abortion (aOR 1.67–11.52, all *P*<0.05). Conversely, educational level of college or higher, household annual income over 80,000 Chinese yuan were associated with lower prevalence of induced abortion and repeat induced abortion (aOR 0.52–0.80, all *P*<0.05). Induced abortion remains noticeable in China. Sustained efforts are required to reduce unintentional pregnancy, improve reproductive health and post-abortion care services, and promote women's health.

## Introduction

During 2015–2019, there were 73.3 million induced abortions each year on average, which corresponded to a global annual rate of 39 abortions per 1,000 women aged 15–49 years (1). Around 45% of all induced abortions are unsafe, of which 97% take place in developing countries (2). As a measure of unplanned pregnancy and unmet contraceptive need, repeated induced abortion also needs growing public concern (3, 4). Moreover, repeated induced abortion is associated with an increased risk of adverse pregnancy outcomes in a subsequent pregnancy (5). Therefore, efforts are needed to reduce unintentional pregnancy and improve reproductive health services.

Reproductive health and induced abortion have attracted international attention (6). The 2030 Agenda for Sustainable Development is committed to ensuring universal access to sexual and reproductive health care services, including family planning, information, and education (7). The Global Strategy for Women's, Children's, and Adolescents' Health (2016–2030) has proposed evidence-based health interventions, which highlight information, counseling, and services for comprehensive sexual and reproductive health (including contraception), as well as safe abortion (wherever legal) and post-abortion care (8).

Induced abortion is widely practiced in China. In 2003, the number of induced abortions in China accounted for a fifth of all abortions worldwide (9). There were 6.1 to 9.9 million induced abortions annually in China between 2000 and 2019 (10). Induced abortion is legal in China since the Regulation of Contraception and Induced Abortion was approved in 1953 (11). Previous strict family planning policy might be responsible for the common induced abortions (12). The one-child policy was introduced in 1979 and ended in 2015 (12). Then, a universal two-child policy was implemented on January 1 2016, and a subsequent brief baby boom was observed from 2015 to 2016 (12, 13). However, the birth rate decreased from 13.57‰ in 2017 to 8.52‰ in 2020 (14). Therefore, China rapidly shifted from the two-child policy to the three-child policy on 31 May 2021, by allowing all couples to have up to three children (15). China attaches great importance on reproductive health and induced abortion. Recently, the Program for the Development of Chinese Women (2021–2030) has proposed to strengthen basic contraceptive services, prevent unwanted pregnancies, and reduce induced abortion for non-medical needs (16).

There are several researches about the prevalence of induced abortion among Chinese women of childbearing age (17–21). However, previous studies were outdated (17, 21), or focused on local areas (17, 18) or some special groups such as married women (17, 21) and migrant population (20). Based on data from three comparable cross-sectional surveys among Chinese women of childbearing age in 2016, 2017, and 2021, this study aimed to estimate the latest prevalence of induced abortion among Chinese women of childbearing age, and explore the related factors, thus helping make targeted policies and measures to reduce unintentional pregnancy, improve reproductive health services, and promote women's health.

## Materials and methods

### Study design and study population

Three comparable cross-sectional surveys of Chinese women of childbearing age were conducted in June 2016, June 2017, and June 2021 using a two-stage stratified sampling method (22) on the largest online survey platform in China: Wen Juan Xing (Changsha Ranxing Information Technology Co., Ltd., Hunan, China). A sample database covering over 2.6 million respondents was established by this online platform, whose personal information was confirmed to ensure an authentic, diverse and representative sample (23). For the sample size calculation, a precision approach was performed, with an estimated prevalence of 18% [as previously reported in China (21)]. Consequently, it was calculated that there is sufficient precision if a minimum of 1,800 women are included in the study with a 95% confidence level and 1.8% margin of error. Those who agreed to participate in these surveys completed the online questionnaires using smartphones. Informed consent was obtained from these participants. We ensured strict quality control and confidentiality of personal privacy in the investigation process through carefully checking the answered questionnaires and keeping every participant's personal information secret.

The inclusion criteria were Chinese women aged 18–49 years in 2016, 2017, and 2021. The exclusion criteria were participants whose abortion information were missing or unreliable. The surveys included 3,647 women in 2016, 6,243 in 2017, and 5,091 in 2021, for a total of 14,981 women, covering 31 provinces in mainland China. After quality control and manual check procedures to exclude ineligible, incomplete, invalid or beyond the age range questionnaires, the final sample consisted of 14,573 respondents (97.28%) involving 3,630 women in 2016, 6,127 in 2017, 4,816 in 2021.

### Data collection

In the three surveys, a similar self-administered questionnaire was designed to collect information from the participants, including sociodemographic characteristics (age, region, ethnicity, residence, educational level, marital status, occupation, and annual household income), reproductive status (number of children), current contraception (whether using contraception currently or not, and whether intend to acquire contraceptive knowledge or not), and times of induced abortion. The primary outcome of the three surveys was the prevalence of induced abortion and repeat induced abortion, defined as the proportion of respondents who self-reported having experienced induced abortion and repeat induced abortion. Repeat induced abortion referred to those reporting at least two previous induced abortion (3). As for abortion, the relevant question was “Have you ever experienced an abortion?” (Answer yes or no). If the answer is “yes,” the participants need to report the times of spontaneous abortion and the times of induced abortion, respectively. From this question, we could acquire the times of induced abortion in every respondent.

### Statistical analysis

We used proportion to describe categorical variables. The prevalence of induced abortion and repeat induced abortion as well as their 95% confidence intervals (CIs) were calculated. χ2 test was used to compare the prevalence in different characteristic groups. Multivariate logistic regression was adopted to examine the factors related to induced abortion and repeated induced abortion. We calculated the adjusted odds ratios (aORs) and their 95%CIs. The related factors included age group, region, ethnicity residence, educational level, marital status, occupation, annual household income, number of children, current contraception, intent to acquire the contraceptive knowledge and survey year. Additionally, we analyzed subgroups stratified by survey year and age. Two-sided *P*-values <0.05 were considered statistically significant. All analyses were performed with R version 4.0.5 (R Project for Statistical Computing).

## Results

### The characteristics of the study population

The 2016, 2017, and 2021 surveys included 3,630, 6,127, and 4,816 women aged 18–49 years, respectively. Of the 14,573 women, 55.06% were 30 years of age or older, 65.11% lived in the eastern region, 96.29% were the Han nationality, 62.20% were urban, 71.10% were married, and 63.12% had children. 89.04% of the respondents were using contraception currently, and 31.85% intended to acquire contraceptive knowledge (Table 1).

**Table 1:** The prevalence of induced abortion and repeated induced abortion among Chinese women aged 18–49 years.

**Characteristics**	***n* (%)**	**Induced abortion**			**Repeat induced abortion**		
		**No of women**	**Prevalence (95%CI)**	**aOR (95%CI)**	**No of women**	**Prevalence (95%CI)**	**aOR (95%CI)**
**Total**	14,573 (100)	2,434	16.70 (16.10–17.31)		1,002	6.88 (6.46–7.29)	
**Survey year**
2016	3,630 (24.91)	680	18.73 (17.46–20.00)	1	282	7.77 (6.90–8.64)	1
2017	6,127 (42.04)	986	16.09 (15.17–17.01)	0.50 (0.44–0.57) *	397	6.48 (5.86–7.10)	0.52 (0.43–0.63) *
2021	4,816 (33.05)	768	15.95 (14.91–16.98)	0.81 (0.71–0.92) *	323	6.71 (6.00–7.41)	0.79 (0.66–0.95) *
**Age group (years)**
18–24	2,763 (18.96)	87	3.15 (2.50–3.80)	1	21	0.76 (0.44–1.08)	1
25–29	3,786 (25.98)	373	9.85 (8.90–10.80)	2.27 (1.73–2.97) *	120	3.17 (2.61–3.73)	3.23 (1.94–5.37) *
30–34	3,631 (24.92)	649	17.87 (16.63–19.12)	3.48 (2.64–4.59) *	232	6.39 (5.59–7.18)	5.28 (3.15–8.83) *
35–49	4,393 (30.14)	1,325	30.16 (28.80–31.52)	6.31 (4.78–8.32) *	629	14.32 (13.28–15.35)	11.52 (6.91–19.21) *
**Region**
Eastern	9,489 (65.11)	1,397	14.72 (14.01–15.44)	1	502	5.29 (4.84–5.74)	1
Central	3,176 (21.79)	609	19.18 (17.81–20.54)	1.36 (1.21–1.52) *	297	9.35 (8.34–10.36)	1.67 (1.42–1.96) *
Western	1,908 (13.09)	428	22.43 (20.56–24.30)	1.72 (1.50–1.98) *	221	11.58 (10.15–13.02)	2.33 (1.94–2.80) *
**Ethnicity**
Han	14,032 (96.29)	2,348	16.73 (16.12–17.35)	1	959	6.83 (6.42–7.25)	1
Others	541 (3.71)	86	15.90 (12.82–18.98)	0.93 (0.72–1.21)	43	7.95 (5.67–10.23)	1.07 (0.76–1.52)
**Residence**
Rural	5,509 (37.80)	788	14.30 (13.38–15.23)	1	310	5.63 (5.02–6.24)	1
Urban	9,064 (62.20)	1,646	18.16 (17.37–18.95)	1.06 (0.94–1.20)	692	7.63 (7.09–8.18)	1.11 (0.93–1.32)
**Educational level**
Junior high school or below	851 (5.84)	234	27.50 (24.50–30.50)	1	114	13.4 (11.11–15.68)	1
Senior high school	2,332 (16.00)	591	25.34 (23.58–27.11)	0.94 (0.77–1.14)	258	11.06 (9.79–12.34)	0.81 (0.62–1.05)
College or higher	11,390 (78.16)	1,609	14.13 (13.49–14.77)	0.59 (0.48–0.72) *	630	5.53 (5.11–5.95)	0.52 (0.40–0.69) *
**Marital status**
Single	4,212 (28.90)	214	5.08 (4.42–5.74)	1	96	2.28 (1.83–2.73)	1
Married	10,361 (71.10)	2,220	21.43 (20.64–22.22)	1.39 (1.14–1.70) *	906	8.74 (8.20–9.29)	0.80 (0.61–1.06)
**Occupation**
Employee	3,569 (24.49)	537	15.05 (13.87–16.22)	1	203	5.69 (4.93–6.45)	1
Factory worker	1,658 (11.38)	270	16.28 (14.51–18.06)	1.01 (0.83–1.22)	105	6.33 (5.16–7.51)	0.99 (0.74–1.32)
Farmer	458 (3.14)	129	28.17 (24.05–32.29)	1.16 (0.88–1.52)	58	12.66 (9.62–15.71)	1.10 (0.76–1.60)
Service staff	1,492 (10.24)	298	19.97 (17.94–22.00)	1.30 (1.07–1.56) *	129	8.65 (7.22–10.07)	1.35 (1.03–1.76) *
Civil servant	3,858 (26.47)	731	18.95 (17.71–20.18)	1.31 (1.13–1.51) *	313	8.11 (7.25–8.97)	1.38 (1.12–1.70) *
Others	3,538 (24.28)	469	13.26 (12.14–14.37)	1.65 (1.39–1.95) *	194	5.48 (4.73–6.23)	1.67 (1.31–2.12) *
**Annual household income**
<30,000 CNY	3,124 (21.44)	518	16.58 (15.28–17.89)	1	230	7.36 (6.45–8.28)	1
30,000–80,000 CNY	5,150 (35.34)	883	17.15 (16.12–18.18)	0.90 (0.79–1.03)	363	7.05 (6.35–7.75)	0.86 (0.71–1.03)
>80,000 CNY	6,299 (43.22)	1,033	16.40 (15.49–17.31)	0.80 (0.70–0.93) *	190	3.02 (2.59–3.44)	0.79 (0.65–0.96) *
**Number of children**
0	5,375 (36.88)	237	4.41 (3.86–4.96)	1	68	1.27 (0.97–1.56)	1
≥1	9,198 (63.12)	2,197	23.89 (23.01–24.76)	2.85 (2.35–3.46) *	934	10.15 (9.54–10.77)	4.06 (2.91–5.65) *
**Current contraception**
Not using	1,597 (10.96)	228	14.28 (12.56–15.99)	1	95	5.95 (4.79–7.11)	1
Using	12,976 (89.04)	2,206	17.00 (16.35–17.65)	1.19 (1.01–1.40) *	907	6.99 (6.55–7.43)	1.11 (0.88–1.40)
**Whether Intent to acquire contraceptive knowledge or not**							
No	9,931 (68.15)	1,640	16.51 (15.78–17.24)	1	650	6.55 (6.06–7.03)	1
Yes	4,642 (31.85)	794	17.10 (16.02–18.19)	1.22 (1.11–1.35) *	352	7.58 (6.82–8.34)	1.42 (1.23–1.63) *

aOR, adjusted odd ratio; 95%CI, 95% confidence interval; CNY, Chinese yuan.

* indicates significant at p-value <0.05.

### The prevalence of self-reported induced abortion and repeat induced abortion

**Figure 1:**
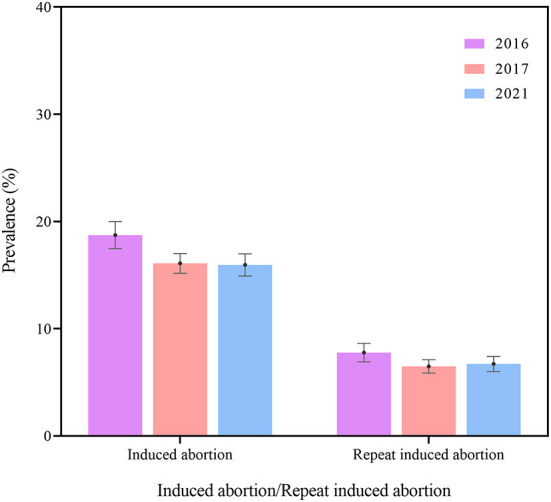
The prevalence of induced abortion and repeat induced abortion among Chinese women in 2016, 2017, and 2021.

Of the 14,573 respondents, 16.70% (95%CI 16.10%–17.31%) self-reported having experienced induced abortion, while 6.88% (95%CI 6.46%–7.29%) self-reported having had repeat induced abortion. The prevalence of induced abortion in 2017 (16.09% vs. 18.73%, *P* < 0.05) and in 2021 (15.95% vs. 18.73%, *P* < 0.05) were lower than that in 2016 (Figure 1, Table 1). The prevalence of induced abortion and repeat induced abortion were higher among women of older ages, living in Central and Western regions, and having children (all *P* < 0.05) (Supplementary Figure 1). Similar tendencies were observed in all 3 years.

### Factors related to induced abortion and repeat induced abortion

Multivariate models showed that the age range of 25–49 years (aOR 2.27–6.31, all *P* < 0.05), living in western (aOR 1.72, 95%CI 1.50–1.98) and central (aOR 1.36, 95%CI 1.21–1.52) regions, being married (aOR 1.39, 95%CI 1.14–1.70), working as service staff (aOR 1.30, 95%CI 1.07–1.56) and civil servants (aOR 1.31, 95%CI 1.13–1.51), having children (aOR 2.85, 95%CI 2.35–3.46), using contraception currently (aOR 1.19, 95%CI 1.01–1.40) and intending to acquire contraceptive knowledge (aOR 1.22, 95%CI 1.11–1.35) were associated with a higher prevalence of induced abortion. The age range of 25–49 years (aOR 3.23–11.52, all *P* < 0.05), living in western (aOR 2.33, 95%CI 1.94–2.80) and central (aOR 1.67, 95%CI 1.42–1.96) regions, working as service staff (aOR 1.35, 95%CI 1.03–1.76) and civil servants (aOR 1.38, 95%CI 1.12–1.70), having children (aOR 4.06, 95%CI 2.91–5.65), and intending to acquire contraceptive knowledge (aOR 1.42, 95%CI 1.23–1.63) were also related to a higher prevalence of repeat induced abortion. In contrast, educational level of college or higher and household annual income over 80,000 Chinese yuan (CNY) were associated with lower prevalence of both induced abortion and repeat induced abortion (aOR 0.52–0.80, all *P* < 0.05) (Table 1). Tables 2, 3 presented the subgroup analysis stratified by survey year and age. The association between induced abortion and factors including age group, region, and having children remained stable.

**Table 2:** Factors related to induced abortion and repeated induced abortion in the multivariate model stratified by survey year.

	**2016**					**2017**					**2021**				
	***n* (%)**	**Induced abortion [% (*n*)]**	**aOR (95%CI)**	**Repeat induced abortion [% (*n*)]**	**aOR (95%CI)**	***n* (%)**	**Induced abortion [% (*n*)]**	**aOR (95%CI)**	**Repeat induced abortion [% (*n*)]**	**aOR (95%CI)**	***n* (%)**	**Induced abortion [% (*n*)]**	**aOR (95%CI)**	**Repeat induced abortion [% (*n*)]**	**aOR (95%CI)**
**Total**	3,630 (100)	18.73 (680)	–	7.77 (282)		6,127 (100)	16.09 (986)	–	6.48 (397)		4,816 (100)	15.95 (768)	–	6.71 (323)	–
**Age group (years)**															
18–24	691 (19.04)	2.46 (17)	1	0.29 (2)	1	556 (9.07)	5.94 (33)	1	1.08(6)	1	1,516 (31.48)	2.44 (37)	1	0.86 (13)	1
25–29	1,118 (30.80)	10.82 (121)	2.24 (1.27–3.96) *	3.13 (35)	6.31 (1.46–27.35) *	1,575 (25.71)	9.59 (151)	1.34 (0.88–2.05)	3.49 (55)	3.26 (1.34–7.92) *	1,093 (22.70)	9.24 (101)	2.30 (1.45–3.64) *	2.74 (30)	1.34 (0.62–2.90)
30–34	835 (23.00)	19.76 (165)	3.14 (1.75–5.64) *	7.90 (66)	12.28 (2.81–53.66) *	1,668 (27.22)	15.71 (262)	1.98 (1.28–3.04) *	5.22 (87)	4.28 (1.74–10.54) *	1,128 (23.42)	19.68 (222)	4.04 (2.53–6.43) *	7.00 (79)	2.50 (1.18–5.32) *
35–49	986 (27.16)	38.24 (377)	6.97 (3.90–12.46) *	18.15 (179)	26.30 (6.04–114.44) *	2,328 (38.00)	23.20 (540)	3.09 (2.01–4.75) *	10.70 (249)	9.32 (3.81–22.82) *	1,079 (22.40)	37.81 (408)	8.01 (5.05–12.72) *	18.63 (201)	6.09 (2.91–12.74) *
**Region**															
Eastern	2,532 (69.75)	15.84 (401)	1	5.45 (138)	1	4,276 (69.79)	14.34 (613)	1	5.24 (224)	1	2,681 (55.67)	14.29 (383)	1	5.22 (140)	1
Central	711 (19.59)	22.93 (163)	1.63 (1.29–2.06) *	11.67 (83)	2.27 (1.66–3.11) *	1,410 (23.01)	19.79 (279)	1.34 (1.14–1.58) *	9.15 (129)	1.62 (1.28–2.05) *	1,055 (21.91)	15.83 (167)	1.21 (0.96–1.51)	6.35 (67)	1.30 (0.94–1.79)
Western	387 (10.66)	29.97 (116)	2.33 (1.76–3.08) *	15.76 (61)	3.31 (2.31–4.74) *	441 (7.20)	21.32 (94)	1.48 (1.15–1.91) *	9.98 (44)	1.76 (1.24–2.51) *	1,080 (22.42)	20.19 (218)	1.70 (1.37–2.11) *	10.74 (116)	2.49 (1.87–3.32) *
**Number of children**															
0	1,506 (41.49)	4.18 (63)	1	1.20 (18)	1	1,639 (26.75)	6.28 (103)	1	1.95 (32)	1	2,230 (46.30)	3.18 (71)	1	0.81 (18)	1
≥1	2,124 (58.51)	29.05 (617)	2.73 (1.90–3.91) *	12.43 (264)	4.16 (2.24–7.74) *	4,488 (73.25)	19.67 (883)	2.83 (2.14–3.76) *	8.13 (365)	3.53 (2.22–5.62) *	2,586 (53.70)	26.95 (697)	2.91 (1.99–4.26) *	11.79 (305)	4.90 (2.46–9.76) *

aOR, adjusted odd ratio; 95%CI. 95% confidence interval.

* indicates significant at p-value <0.05.

aOR was adjusted for age group, region, residence, educational level, marital status, occupation, annual household income, number of children, current contraception and intent to acquire contraceptive knowledge.

**Table 3:** Factors related to induced abortion and repeated induced abortion in the multivariate model stratified by age group.

	**18–29 years**					**30–34 years**					**35–49 years**				
	***n* (%)**	**Induced abortion [% (*n*)]**	**aOR (95%CI)**	**Repeat induced abortion [% (*n*)]**	**aOR (95%CI)**	***n* (%)**	**Induced abortion [% (*n*)]**	**aOR (95%CI)**	**Repeat induced abortion [% (*n*)]**	**aOR (95%CI)**	***n* (%)**	**Induced abortion [% (*n*)]**	**aOR (95%CI)**	**Repeat induced abortion [% (*n*)]**	**aOR (95%CI)**
**Total**	6,549 (100)	7.02 (460)	–	2.15 (141)	–	3,631 (100)	17.87 (649)	–	6.39 (232)	–	4,393 (100)	30.16 (1,325)	–	14.32 (629)	–
**Survey year**															
2016	1,809 (27.62)	7.63 (138)	1	2.05 (37)	1	835 (23.00)	19.76 (165)	1	7.90 (66)	1	986 (22.44)	38.24 (377)	1	18.15 (179)	1
2017	2,131 (32.54)	8.63 (184)	0.71(0.54–0.94) *	2.86 (61)	0.96 (0.59–1.57)	1,668 (45.94)	15.71 (262)	0.51 (0.39–0.66) *	5.22 (87)	0.40 (0.27–0.59) *	2,328 (52.99)	23.20 (540)	0.44 (0.36–0.53) *	10.70 (249)	0.51 (0.40–0.65)
2021	2,609 (39.84)	5.29 (138)	0.67 (0.51–0.87)	1.65 (43)	0.80 (0.50–1.28)	1,128 (31.06)	19.68 (222)	0.85 (0.67–1.08)	7.00 (79)	0.69 (0.48–0.99) *	1,079 (24.56)	37.81 (408)	0.90 (0.75–1.09)	18.63 (201)	0.90 (0.71–1.14) *
**Region**															
Eastern	4,091 (62.47)	6.29 (257)	1	1.78 (73)	1	2,501 (68.88)	14.83 (371)	1	4.28 (107)	1	2,897 (65.95)	26.54 (769)	1	11.11 (322)	1
Central	1,475 (22.52)	7.25 (107)	1.19 (0.93–1.53)	2.03 (30)	1.12 (0.72–1.75)	720 (19.83)	21.11 (152)	1.36 (1.09–1.69) *	9.03 (65)	1.94 (1.39–2.70) *	981 (22.33)	35.68 (350)	1.42 (1.21–1.68) *	18.76 (184)	1.73 (1.41–2.12) *
Western	983 (15.01)	9.77 (96)	1.71 (1.30–2.26) *	3.87 (38)	2.21 (1.41–3.44) *	410 (11.29)	30.73 (126)	2.13 (1.65–2.75) *	14.63 (60)	3.08 (2.14–4.42) *	515 (11.72)	40.00 (206)	1.51 (1.23–1.86) *	23.88 (123)	2.09 (1.63–2.68) *
**Number of children**															
0	4,431 (67.66)	3.02 (134)	1	0.74 (33)	1	603 (16.61)	9.12 (55)	1	2.49 (15)	1	341 (7.76)	14.08 (48)	1	5.87 (20)	1
≥1	2,118 (32.34)	15.39 (326)	2.28 (1.71–3.06) *	5.10 (108)	2.85 (1.62–5.00) *	3,028 (83.39)	19.62 (594)	2.40 (1.68–3.43) *	7.17 (217)	4.51 (2.34–8.68) *	4,052 (92.24)	31.52 (1,277)	2.70 (1.93–3.79) *	15.03 (609)	2.93 (1.80–4.79) *

aOR, adjusted odd ratio; 95%CI, 95% confidence interval.

* indicates significant at p-value <0.05.

aOR was adjusted for survey year, region, residence, educational level, marital status, occupation, annual household income, number of children, current contraception and intent to acquire contraceptive knowledge.

## Discussion

This study found that of the 14,573 Chinese women aged 18–49 years, 16.70% self-reported having experienced induced abortion, while 6.88% self-reported having had repeat induced abortion. Among other countries where induced abortions were legal, the prevalence of induced abortion was 22% in France (24), 21.1% in Nepal (25), and 10.25% in Australia (26), while the prevalence of repeat induced abortion was 8% in France (24) and 11.9% in the United Kingdom (27). In previous studies among Chinese women, the prevalence of induced abortion was 8.13–24.0% (17, 19, 21), and the prevalence of repeat induced abortion was 0.8–11.7% (17, 28), slightly different from our results. This discrepancy between these studies might be attributed to the different characteristics of the study population. Our study population was Chinese women of childbearing age, while others focused on women in a local area of China or specific groups such as unmarried women. Moreover, these studies were not conducted at the same time. However, following China's new three-child policy, reproductive health and induced abortion remain noticeable and need more attention.

Our study also showed that the prevalence of induced abortion in 2017 and 2021 were lower than that in 2016. This might result from the gradually relaxed fertility policy (12, 15), which reduced the prevalence of induced abortion through childbearing (21). Furthermore, both global (1) and domestic (21) researches suggested that the decreasing trend was slowing down, potentially resulting from people's gradually satisfied fertility intention and the increasing use of less effective short-acting contraceptive methods among Chinese women (29). Although China's new three-child policy could lead to a further decline, there are great numbers of women of childbearing age who have unintentional pregnancies and subsequent induced abortions on account of the large population in China. Therefore, sustained efforts are still needed to improve reproductive health and post-abortion care services.

As for relevant sociodemographic factors, we found that older women had higher prevalence of induced abortion and repeat induced abortion, consistent with previous researches (18, 19). This reflects the longer exposure to sexual intercourse and thereby increased risks of unintended pregnancies and subsequent induced abortions for older women (18). It is essential to promote contraceptive methods, and provide safe abortion and post-abortion care for these women. However, induced abortion among young women should not be ignored. A national survey covering 11,076 unmarried young women aged 15–24 years showed that the self-reported prevalence of induced abortion was 3.9% and the prevalence of repeat induced abortion was 0.8% (28). Moreover, adolescents' sexual and reproductive knowledge is limited due to the slow progress of education (30). The 2009 Survey of Youth Access to Reproductive Health in China showed that most unmarried young people aged 15–24 years sought sexual and reproductive knowledge from books and the Internet, while more than 50% of their demand for reproductive health knowledge, contraceptive methods, sexually transmitted disease treatment, and safe abortion was not fulfilled (30). Additionally, reproductive health services and education in China traditionally target married individuals, with limited contraceptive services and counseling available for unmarried women (31). Therefore, measures are required to improve reproductive health knowledge, provide accessible services, and reduce unintentional pregnancy among unmarried young women and adolescents.

We also found that women living in western and central regions, and having children were more likely to have experienced induced abortion and repeat induced abortion, while those with a higher educational level and a higher annual household income were less likely, consistent with previous studies (17, 19, 21, 32). The higher prevalence in western and central regions might be ascribed to the strict implementation of family planning policy and a strong preference for male children (21). Furthermore, because of poor medical conditions in these areas, the problem of unsafe induced abortion is more noteworthy. Women with children were more likely to have induced abortions due to satisfied fertility intention, and postpartum unintentional pregnancy and induced abortion should not be neglected. A cohort study conducted at 60 public hospitals in China indicated that the cumulative rates of unintended pregnancy and induced abortion within 24 months of delivery were 13.1% and 10.4%, respectively (33). Postpartum care is supposed to highlight contraceptive counseling and services. Meanwhile, those without children deserved equal concern because of their possibility of childbearing in the future. For the relationship between educational level, annual household income and induced abortion, a possible explanation is that women with higher educational levels and income might have higher levels of health literacy, especially better contraceptive knowledge and practice which allow them to better avoid unintended pregnancies and ensuing abortions (18, 34). In short, we should attach importance to women living in western and central regions, with and without children, and with a lower educational level or household income.

For contraception, our results suggested that women using contraception currently had a higher prevalence of induced abortion. This could be partially explained by the increasing awareness of the importance of contraception among women who have a history of induced abortion. Another possible reason is that contraceptive use and abortion share a common cause: a strong desire to avoid having a child. We also found that 89.04% of Chinese women were using contraception currently, similar to previous surveys (35). Despite the high prevalence of contraception in China, large numbers of induced abortions still existed, calling for more attention to inappropriate contraceptive use and contraceptive failure (36). Moreover, there was a decreasing trend in the prevalence of contraception among married women recently, from 89.1% in 2010 to 80.6% in 2018 (35). Furthermore, with the relaxation of fertility policy, the prevalence of long-acting contraceptive use decreased from 80.0% in 2010 to 63.6% in 2018, while the prevalence of short-acting contraceptive use increased from 9.1% in 2010 to 17.0% in 2018 (37). The short-acting contraceptive methods are less effective than the long-acting methods (17). Additionally, our study showed that women intending to acquire contraceptive knowledge were more likely to have a history of induced abortion. These women might have experienced unintentional pregnancies and subsequent induced abortions due to a lack of knowledge, thereby becoming more willing to know about contraception. Therefore, it is necessary to promote sexual and reproductive education, provide sufficient counseling and effective methods, and avoid contraceptive failure.

The main strength of our study is that it is the first to estimate the prevalence of induced abortion among Chinese women of childbearing age, and explore the related factors based on the latest sample covering 31 provinces in mainland China. It could provide scientific evidence for making targeted policies and measures to reduce unintentional pregnancy, improve reproductive health and post-abortion care services, and safeguard women's health. However, there are some limitations. First, women who were not internet users were not involved in our study, possibly leading to a highly educated sample. Nevertheless, there were 989 million internet users in China by December 2020, and 99.7% of them surf the internet by mobile phone (38). Second, some women might under-report induced abortions due to this sensitive topic, even though we ensured strict quality control and confidentiality of personal privacy in the investigation process (39).

To summarize, induced abortion remains noticeable in China. More attention should be paid to women living in western and central regions, and with a lower educational level or household income. In the future, sustained efforts are required to make targeted and feasible measures to reduce unintentional pregnancy, improve reproductive health and post-abortion care services, and promote women's health.

## Data availability statement

The data analyzed in this study is subject to the following licenses/restrictions: The datasets generated and/or analyzed for this study are not publicly available due to intellectual property and confidentiality concerns, but are available from the corresponding author on reasonable request. Requests to access these datasets should be directed to ML, liumin@bjmu.edu.cn.

## Ethics statement

The studies involving human participants were reviewed and approved by Institutional Review Board of the Chinese Association of Maternal and Child Health Studies with approval number CAMCHS16001. The patients/participants provided their online informed consent to participate in this study.

## Author contributions

LK searched the literature, analyzed the data, interpreted the results, and drafted the manuscript. WJ, QM, and YW collected the data. SZ and JL revised the manuscript. ML conceived the study, designed the study, supervised the study, interpreted the results, and revised the manuscript. All authors read and approved the final manuscript.

## Funding

This study was supported by the grant from National Natural Science Foundation of China [grant numbers 71934002 and 72122001] and National Key Research and Development Project of China [grant numbers 2021ZD0114101, 2021ZD0114104, and 2021ZD0114105]. The funders had no role in study design, data collection and analysis, decision to publish, or preparation of the paper. No payment was received by any of the co-authors for the preparation of this article. The corresponding author had full access to all the data in the study and had final responsibility for the decision to submit for publication.

## Conflict of interest

The authors declare that the research was conducted in the absence of any commercial or financial relationships that could be construed as a potential conflict of interest.

## Publisher's note

All claims expressed in this article are solely those of the authors and do not necessarily represent those of their affiliated organizations, or those of the publisher, the editors and the reviewers. Any product that may be evaluated in this article, or claim that may be made by its manufacturer, is not guaranteed or endorsed by the publisher.
