# Supplementary material for: Prevalence of induced abortion among Chinese women aged 18–49 years: Findings from three cross-sectional studies
Source: Front Public Health. 2022 Oct 3;10:926246. doi: 10.3389/fpubh.2022.926246 (PMC9575936; doi:10.3389/fpubh.2022.926246)
Supplement: Supplementary file 1 [file Table_1.DOCX]

Supplementary Material

# Supplementary Figure


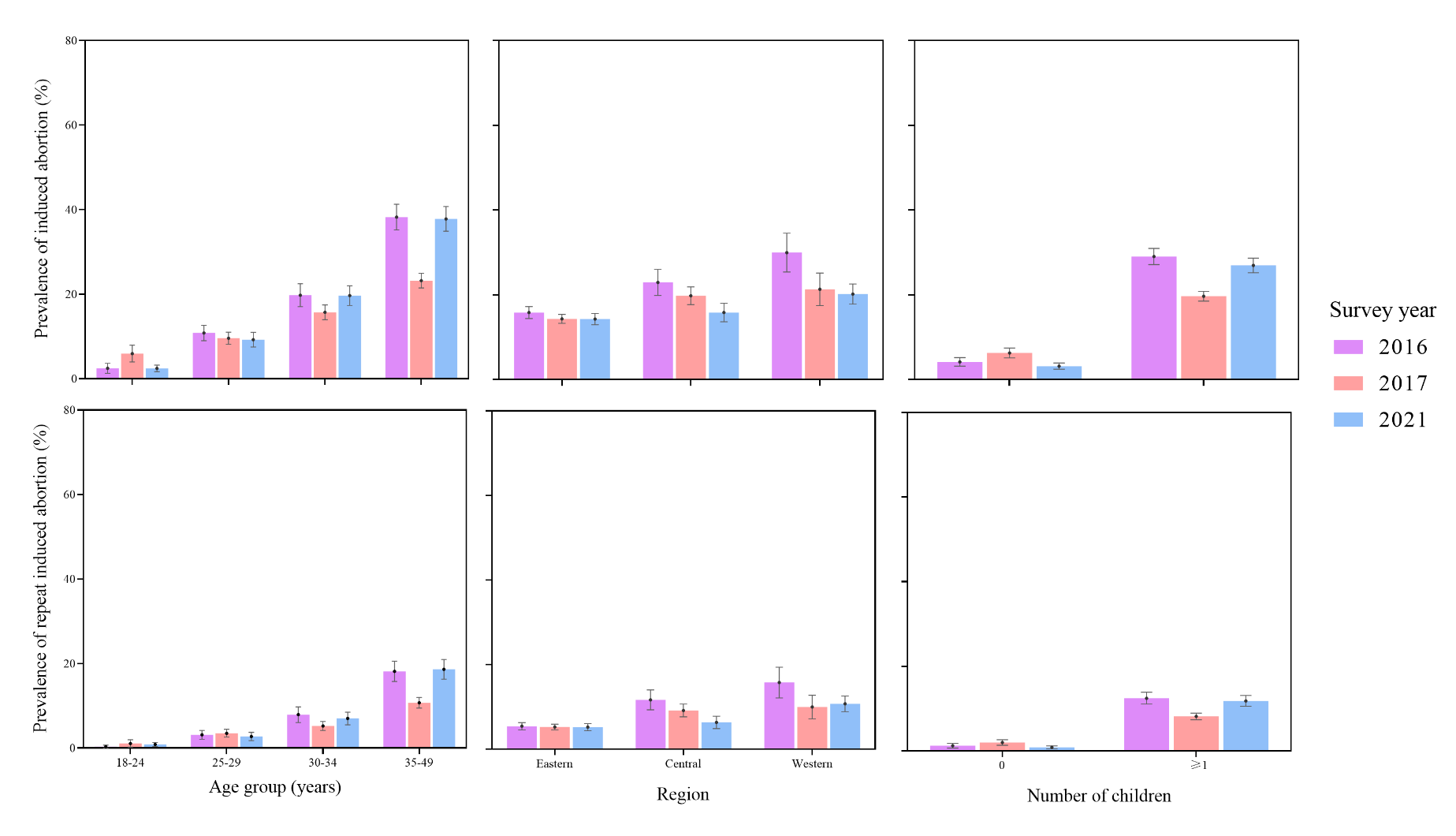


**Supplementary Figure 1.** The prevalence of induced abortion and repeat induced abortion among Chinese women of different age groups, regions, and numbers of children in 2016, 2017 and 2021
